# Association between PD-1 single nucleotide gene variants and the risk of metastatic melanoma

**DOI:** 10.1007/s00403-024-03034-9

**Published:** 2024-06-16

**Authors:** Andrea Boutros, Roberta Carosio, Dalila Campanella, Barbara Banelli, Anna Morabito, Maria Pia Pistillo, Elena Croce, Paola Queirolo, Enrica Teresa Tanda, Edoardo Raposio, Vincenzo Fontana, Francesco Spagnolo

**Affiliations:** 1 Skin Cancer Unit, Medical Oncology 2, IRCCS Ospedale Policlinico San Martino, Genova, Italy; 2 Department of Internal Medicine and Medical Specialties (DIMI), School of Medicine, University of Genoa, Genova, Italy; 3 Tumor Epigenetics Unit, IRCCS Ospedale Policlinico San Martino, Genova, Italy; 4 Clinical Epidemiology Unit, IRCCS Ospedale Policlinico San Martino, Genova, Italy; 5 Medical Oncology, Ospedale Santa Corona, 17027 Pietra Ligure, Savona, Italy; 6 Division of Melanoma Sarcoma and Rare Tumors, IRCCS European Institute of Oncology, Milan, Italy; 7 Department of Surgical Sciences and Integrated Diagnostics (DISC), Plastic Surgery Division, University of Genoa, Genova, Italy

**Keywords:** Melanoma, SNV, SNP, Single nucleotide gene polymorphism, Cancer risk, PD-1

## Abstract

Previous studies showed an association between single nucleotide gene variants (SNVs) of PD-1 and cancer susceptibility. We analyzed PD1.5 C > T and PD1.7 T > C SNVs to investigate their association with the risk of developing metastatic melanoma (MM). Utilizing a cohort of 125 MM patients treated with anti-PD-1 agents and 84 healthy controls, we examined genotype/allele frequencies through a modified Poisson regression model, adjusted for age and sex. Our findings indicate that the PD1.5 T allele is associated with a reduced risk of MM, showing a significantly lower risk in both codominant (RR = 0.56, 95%CL: 0.37–0.87) and dominant (RR = 0.73 95%CL: 0.59–0.90) models. Conversely, the PD1.7 C allele is linked to an increased risk of MM, with the C/C genotype exhibiting a higher risk in the codominant (RR = 1.65, 95%CL: 1.32–2.05) and allelic (RR = 1.23, 95%CL: 1.06–1.43) models. These results are consistent with previous meta-analyses on other cancer types, mainly highlighting the PD1.5 SNV’s potential role in promoting anti-tumor immunity through increased PD1-positive circulating effector T cell activity.

## Introduction

Several studies have associated single nucleotide gene variants (SNVs) of PD-1 and PD-L1 with cancer susceptibility. Specifically, PD1.5 C > T (rs2227981), as well as PD-L1 G > C (rs4143815) have been associated with a decreased risk of cancer [1, 2]. However, PD1.3 G > A (rs11568821) and PD-1.7 T > C (rs7421861) have been linked to an increased risk of cancer [1, 2]. These findings are supported by the role of PD-1 in regulating T cell functions and tumor-specific immunity [3]. In a previous study including 125 MM patients treated with an anti-PD1 agent, we highlighted the predictive role of PD1.5 C > T, PD1.7 T > C and PD-L1 + 8293 C > A SNVs on the occurrence of immune-related adverse events (irAEs), with PD1.7 SNV also showing a prognostic value [4].

We here report the significant association of PD1.5 and PD1.7 SNVs with the risk of MM in the same patient cohort by comparing genotype/allele frequencies in melanoma patients and 84 healthy control subjects.

## Methods

In the present study, we included 125 patients with advanced melanoma receiving treatment with an anti-PD-1 agent, between January 1st 2013 and December 31st 2020, at the IRCCS Ospedale Policlinico San Martino in Genova, Italy. Control subjects consisted of 84 unrelated healthy blood donors recruited from the Transfusion Medicine Department of the same institution, with their informed consent obtained for DNA analysis [4]. We analyzed five PD-1 SNVs, PD1.3 G > A (rs11568821), PD1.5 C > T (rs2227981), PD1.6 G > A (rs10204525), PD-1.7 T > C (rs7421861) PD1.10 C > G (rs5582977) and three PD-L1 SNVs, PD-L1 + 8293 C > A (rs2890658), PD-L1 C > T (rs2297136) and PD-L1 G > C (rs4143815). Genotyping of DNA extracted from peripheral blood was performed by pyrosequencing (PSQ) or by real-time polymerase chain reaction (PCR) methods [4].

A modified Poisson regression analysis was applied to SNV data stratified according to codominant, dominant, recessive, and allelic models in both groups in order to estimate the relative risk (RR) of MM, along with the corresponding 95% confidence limits (95%CL), adjusted for age at recruitment and sex [5]. All genotype frequencies were preliminarily tested for the Hardy-Weinberg equilibrium (HWE) and no deviations were found (Table 1).

**Table 1:** Codominant, dominant, recessive and allelic models for PD1.5 C > T and PD1.7 T > C SNVs estimated through a modified Poisson regression modelling adjusted for age and sex. Distribution of SNV genotypic and allelic frequencies in 125 patients with advanced melanoma receiving treatment with an anti-PD-1 agent and in 84 healthy blood donors used as controls

Genotypicmodels	*N* = 125 Patients *n* (%)	*N* = 84 Controls *n* (%)	Patients vs. Controls		
			RR	95%CL	*P*-value
PD1.5 C > T(rs2227981)					
**Codominant**					0.011
C/C	51 (40.8)	18 (21.4)	1.00	(Ref.)	
C/T	61 (48.8)	46 (54.8)	0.80	0.65–0.99	
T/T	13 (10.4)	20 (23.8)	0.56	0.37–0.87	
***HWE***	P*=0.399	P*=0.380			
**Dominant**					0.003
C/C	51 (40.80)	18 (21.43)	1.00	(Ref.)	
C/T + T/T	74 (59.20)	66 (78.57)	0.73	0.59–0.90	
**Recessive**					0.036
C/C + C/T	112 (89.60)	64 (76.19)	1.00	(Ref.)	
T/T	13 (10.40)	20 (23.81)	0.63	0.42–0.98	
**Allelic**					0.003
C	163 (65.20)	82 (48.81)	1.00	(Ref.)	
T	87 (34.80)	86 (51.19)	0.78	0.65–0.91	
**PD1.7 T > C(rs7421861)**					
**Codominant**					< 0.001
T/T	59 (47.20)	50 (59.5)	1.00	(Ref.)	
T/C	48 (38.40)	32 (38.1)	1.06	0.83–1.36	
C/C	18 (14.40)	2 (2.4)	1.65	1.32–2.05	
***HWE***	P*=0.119	P*=0.229			
**Dominant**					0.128
T/T	59 (47.20)	50 (59.52)	1.00	(Ref.)	
T/C + C/C	66 (52.80)	34 (40.48)	1.18	0.95–1.47	
**Recessive**					0.077
T/C + T/T	107 (85.60)	82 (97.62)	1.00	(Ref.)	
C/C	18 (14.40)	2 (2.38)	1.58	0.95–2.64	
**Allelic**					0.007
T	166 (66.40)	132 (78.57)	1.00	(Ref.)	
C	84 (33.60)	36 (21.43)	1.23	1.06–1.43	

SNVs: single nucleotide variants;RR: relative risk; 95%CL: 95% confidence limits for RR; P-value: probability level associated with the likelihood ratio test result; Ref.: reference genotype category;HWE:Hardy-Weinberg equilibrium; P*:probability level associated with the chi-square test for departure from the HWE [4]

## Results

We present here key findings from our exploratory investigation into the genetic basis of MM susceptibility. These results were derived from the analysis of 125 melanoma patients included in our previous study and a total of 84 healthy volunteer donors [4].

**Fig. 1:**
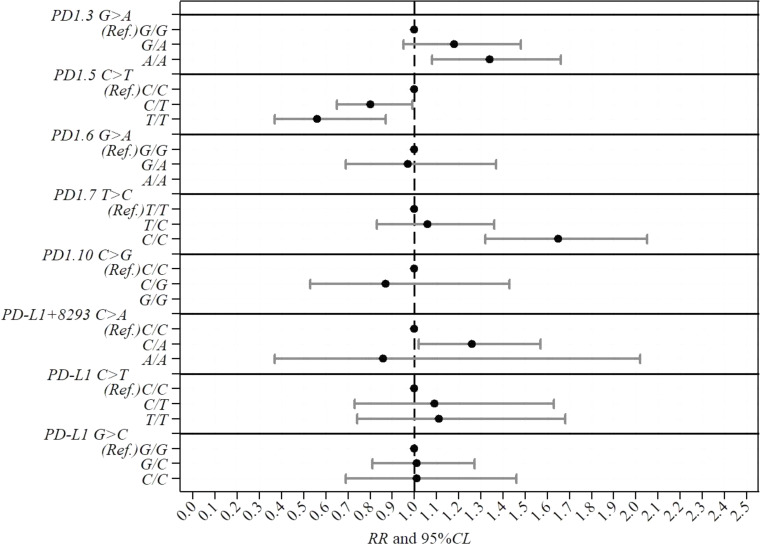
Caterpillar plot illustrating the relative risk (RR) point estimates of metastatic melanoma occurrence along with the corresponding 95% confidence limits (95%CL) for specific single nucleotide gene variants (SNVs), also known as single-nucleotide polymorphisms (SNPs) within the PD-1 and PD-L1 genes according to the codominant model. *Note* Each line corresponds to a SNV comparison between the variant and the reference genotype category (Ref.), with the variant genotypes shown on the left. The black dots represent the RR point estimates while the horizontal lines indicate the 95%CLs. A RR point estimate positioned to the right/left of the dashed vertical line at RR = 1 suggests an increased/decreased risk associated with the variant genotype

In the codominant model, the PD1.5 T/T genotype showed a significantly reduced risk of MM occurrence (RR = 0.56, 95%CL: 0.37–0.87, p-value = 0.011) compared to the reference C/C genotype (Table 1; Fig. 1). The dominant model echoed this protective effect, with combined C/T + T/T genotypes showing a lower risk (RR = 0.73, 95%CL: 0.59–0.90, p-value = 0.003). Allelic modeling reinforced these observations, indicating a lower risk associated with the T allele (RR = 0.78, 95% CL: 0.65–0.91, p-value = 0.003) (Table 1; Fig. 1).

On the other hand, in the codominant model, the PD1.7 C/C genotype exhibited a higher risk (RR = 1.65, 95%CL: 1.32–2.05, p-value < 0.001) compared to the T/T genotype (Table 1; Fig. 1). The recessive model further supported this association, with the C/C genotype presenting a remarkable elevated risk (RR = 1.58, 95% CL: 0.95–2.64, p-value = 0.077). Allelic modeling reinforced the significance, showing an increased risk associated with the C allele (RR = 1.23, 95% CL: 1.06–1.43, p-value = 0.007) (Table 1; Fig. 1).

No significant association was found between MM occurrence and PD1.3 G > A(rs11568821), PD1.6 G > A (rs10204525), PD1.10 C > G (rs5582977), PD-L1 + 8293 C > A (rs2890658), PD-L1 C > T (rs2297136) and PD-L1 G > C (rs4143815) SNVs in all genetic models (Fig. 1).

## Discussion

Our results on PD1.5 and PD1.7 SNV association with MM susceptibility are strongly consistent with what has been observed in previous meta-analyses in other cancer types [1, 2].To our knowledge, no associations of PD-1 SNVs with melanoma risk have been reported so far [6].

The PD1.5 SNV shows a synonymous C > T substitution in exon 5 of *PDCD1* gene and is probably in linkage disequilibrium with other PD-1 gene SNVs responsible for a higher frequency and activity of PD1-positive circulating effector T cells thus promoting robust anti-tumor immune activation [6, 7].

The PD1.7 T > C variant is located in intron 1 of *PDCD1* gene where numerous regulatory and splicing control elements exist but its effects on PD-1 protein expression have not been clearly demonstrated [7].This SNV may disrupt the normal splicing process and alter mRNA secondary structure, potentially leading to modified gene expression and translation inhibition [7]. Our previous study indicates that the presence of the C allele may exhibit a trend for a protective role in irAE onset and, in the homozygous C/C genotype, significantly reduce the risk of death in advanced melanoma patients [4]. This could be attributed to the PD1.7 SNV’s effect in reducing PD-1 expression, directly associated with more efficient antitumor T-cell immunity. However, this effect might be present only in case of exposure to anti-PD-1 agents, or representing, on the other hand, an independent factor in cancer susceptibility in the general population. A critical limitation of our study is the exclusive focus on MM cases, which may slightly affect the generalizability of our findings. Consequently, the associations we have described here are specific to MM patients and probably could not be extended to the general melanoma susceptibility, especially when compared to the representative sample of the general population (healthy donors). In fact, the majority of melanoma cases are diagnosed in a localized or early stage. This clarification allows us to hypothesize that certain genetic profiles may influence the progression rate of melanoma rather than its initial occurrence.

Thus, our results extend our previous findings on the diverse roles of PD-1 SNVs in the context of MM. However, further studies will be needed in larger and different populations to evaluate the relationship between PD-1 and PDL-1 SNVs and MM risk.

## Data Availability

No datasets were generated or analysed during the current study.
